# Sustained complete response of hepatocellular carcinoma with portal vein tumor thrombus following discontinuation of sorafenib: A case report

**DOI:** 10.3892/ol.2013.1664

**Published:** 2013-11-07

**Authors:** KAZUE SHIOZAWA, MANABU WATANABE, TAKASHI IKEHARA, YASUSHI MATSUKIYO, MICHIO KOGAME, MASAHIRO KANAYAMA, TEPPEI MATSUI, YOSHINORI KIKUCHI, KOJI ISHII, YOSHINORI IGARASHI, YASUKIYO SUMINO

**Affiliations:** Division of Gastroenterology and Hepatology, Department of Internal Medicine, Toho University Medical Center, Omori Hospital, Ota-ku, Tokyo 143-8541, Japan

**Keywords:** hepatocellular carcinoma, sorafenib, complete response, portal vein tumor thrombus

## Abstract

Hepatocellular carcinoma (HCC) is the third most common cause of cancer-associated mortality worldwide. No effective treatment has been established for unresectable advanced HCC, and the prognosis is poor. Sorafenib is an oral multi-targeted tyrosine kinase inhibitor for unresectable advanced HCC that significantly improves progression-free and overall survival. However, in the two large phase III clinical trials (the SHARP and Asia-Pacific trials), no cases of complete response (CR) were reported. The present study reports the case of a 68-year-old male with hepatitis C virus-related cirrhosis and multiple recurrent HCCs, with a tumor thrombus of the third portal vein following resection. The patient received 400 mg once daily (half the standard dose) of sorafenib for two years and achieved a CR. At the most recent follow-up examination at one year after the cessation of treatment, the patient was observed to be in remission without clinical or imaging evidence of disease recurrence.

## Introduction

Hepatocellular carcinoma (HCC) is the third most common cause of cancer-associated mortality worldwide (1). Local treatments, including surgical resection and radiofrequency ablation (RFA) for early-stage HCC, give favorable outcomes, but no effective treatment has been established for advanced HCC that is not amenable to surgical resection, and the prognosis of advanced HCC is poor.

Sorafenib (Nexvar; Bayer Healthcare pharmaceuticals; Leverkusen, Germany) is an oral multi-targeted tyrosine kinase inhibitor that is indicated for unresectable advanced HCC and significantly improves progression-free survival (PFS) and overall survival (OS) (2,3). In the SHARP (Sorafenib HCC Assessment Randomized Protocol) trial (2), survival time was significantly prolonged from 7.9 months in the placebo group to 10.7 months in the sorafenib group, but a complete response (CR) was not achieved in any of the 299 patients in the sorafenib group. Similarly, a CR did not occur in any of the 150 patients in the Asia-Pacific trial (conducted in the Asia-Pacific region) (3), indicating that achieving a CR is infrequent in treatment with sorafenib.

The acquisition of a CR following sorafenib treatment has occasionally been reported, and the discontinuation of medication subsequent to acquiring a CR in these cases would be beneficial, as sorafenib is an expensive drug and has adverse effects (4). However, it is unclear whether CR is maintained following discontinuation. The present study describes a case of recurrent HCC with a portal vein tumor thrombus (PVTT) of the third portal vein after resection in a patient who was treated with sorafenib and achieved a CR, which was then maintained for more than one year following the discontinuation of the medication. A literature review is also presented. Written informed consent was obtained from the patient.

## Case report

**Figure 1:**
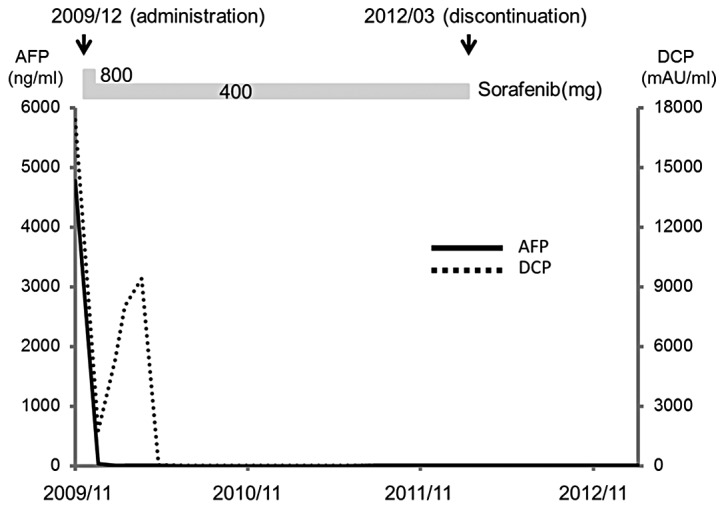
Changes in AFP and DCP levels. The duration of treatment with sorafenib is indicated by the gray bar. The administration of sorafenib resulted in a significant reduction in serum AFP and DCP levels. AFP, α-fetoprotein; DCP, des-γ carboxyprothrombin.

**Figure 2:**
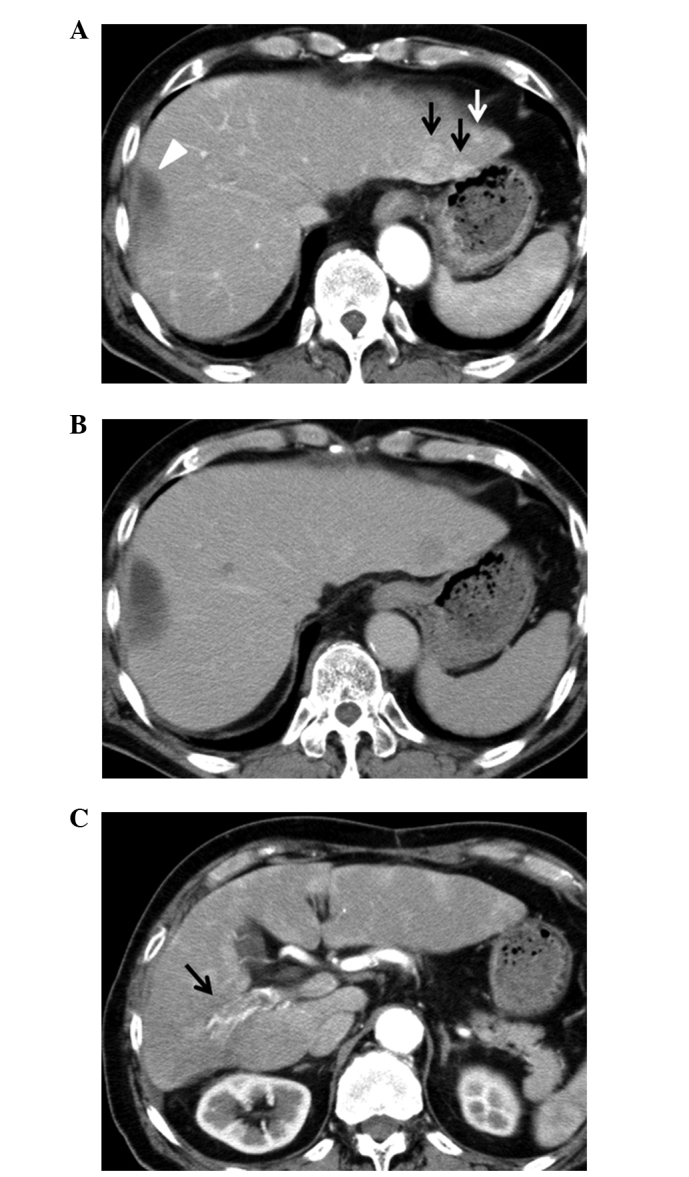
Dynamic computed tomography (CT) prior to treatment. (A) Arterial phase and (B) equilibrium phase showing several hepatocellular carcinomas (arrows) in the left hepatic lobe and biloma (arrow head) by the segmentectomy in S5 liver surface. (C) Arterial phase showing portal vein tumor thrombus (arrow) in the right portal branch.

The patient was a 68-year-old male with hepatitis C virus-related liver cirrhosis. A giant HCC was detected and an S7/S8 segmentectomy of the liver was performed at another hospital. Recurrence in the residual liver, PVTT in the right portal branch and right abdominal disseminated lesions were noted four months after the surgery, although only the disseminated lesions were surgically excised at the request of the patient. The patient was referred to Toho University Medical Center, Omori Hospital (Tokyo, Japan) to continue treatment for the intrahepatic recurrence. In the initial blood tests at the hospital, liver function was graded as Child-Pugh A and tumor marker levels were high: α-fetoprotein (AFP), 4,773 ng/ml; AFP-L3, 60.5%; and des-γ carboxyprothrombin (DCP), 17,400 mAU/ml (Fig. 1). Abdominal computed tomography (CT) showed numerous tumors in the bilateral lobes and a PVTT in the right portal branch (Fig. 2). Oral sorafenib therapy was initiated at the recommended dose of 800 mg/day. Grade 3 hand-foot syndrome (Common Terminology Criteria for Adverse Events version 4.0) (5) developed 7 days after the initiation of sorafenib treatment, and the dose was reduced to 400 mg/day on day 10.

After one month of administration, the AFP level was decreased to 45.7 ng/ml, but there were no changes in PVTT or in the multiple tumors in the bilateral lobes on abdominal CT. The condition was judged to be of a stable disease based on the modified Response Evaluation Criteria in Solid Tumors (mRECIST) (6). A partial response was achieved after six months. On abdominal CT after two years of sorafenib administration, multiple tumors in the bilateral lobes had shrunk and the intense staining due to the PVTT had been resolved, based on which the condition was judged to have achieved a CR. Sorafenib at 400 mg/day was continued thereafter, but mild cerebellar infarction developed at two years and four months after the initiation of administration, and sorafenib was withdrawn at the request of the patient. A CR was maintained for approximately one year after the discontinuation based on abdominal CT findings and normal tumor marker levels.

## Discussion

Sorafenib is a multikinase inhibitor with reported activity against Raf-1, B-Raf, vascular endothelial growth factor receptor 2 (VEGFR2), platelet-derived growth factor receptor (PDGFR) and c-Kit receptors, as well as other receptor tyrosine kinases and serine threonine kinases (7). Sorafenib is a molecular-targeted drug that exerts an antitumor effect by inhibiting tumor growth and vascularization. The efficacy of sorafenib has been shown in the SHARP (2) and Asia-Pacific trials (3). Survival was significantly prolonged in the sorafenib group compared with the placebo group in all these studies, although none of the patients (449 in total) achieved a CR in a RECIST-based judgment of the effect. An evaluation of tumor hemodynamics is now considered to be important for the judgment of therapeutic effect based on the characteristics of the antitumor effect of sorafenib, and the utility of hemodynamic evaluation using mRECIST and contrast-enhanced ultrasonography (CEUS) has previously been described (8). Therefore, the judgment of the therapeutic effect of sorafenib using RECIST in previous clinical studies may not be completely reliable, although it is clear that a CR is rarely achieved with sorafenib treatment.

Certain HCC patients worldwide have been observed to achieve a CR with sorafenib, such as the present case (4,9–12). In this present case, administration was started at 800 mg/day, but the dose was reduced to 400 mg/day soon after initiation due to adverse effects. The recommended dose of sorafenib is 800 mg/day and most reported CR cases have received oral administration at this dose (9,11,12), although Wang *et al*(10) and Inuzuka *et al*(4) have described cases treated with 400 mg/day in which a CR was achieved. These results indicate that further investigation of the usefulness of a low-dose administration of sorafenib may be necessary. It is also of note that the present case had PVTT, since it is considered that an effect with sorafenib is not readily obtained in cases with PVTT. However, Wang *et al*(10) and Sacco *et al*(12) have reported CR in cases with PVTT following treatment with sorafenib. VEGF is important in the vascularization and progression of PVTT in HCC, and sorafenib may have a favorable therapeutic effect on PVTT through the inhibition of the VEGF pathway (13). More detailed investigations of VEGF levels in individual patients may enable a prediction of the efficacy of sorafenib for cases with PVTT prior to treatment.

The most important point in the present case is the maintenance of a CR following the discontinuation of sorafenib. Four cases with the maintenance of a CR subsequent to discontinuation have been reported, including that of the present patient (4,9,10). Wang *et al*(10) described a case with PVTT in which a CR was achieved at a low dose of sorafenib, similar to the present case. A CR was acquired at eight months after the initiation of oral administration and the drug was withdrawn subsequent to achieving a CR, with no recurrence for 16 months after discontinuation. So *et al*(9) reported a case in which sorafenib was used at the recommended dose for HCC with lung metastasis. A CR was achieved following five months of oral administration and there was no recurrence for six months after discontinuation. Inuzuka *et al*(4) also reported achieving a CR in a case of HCC with lung metastasis treated with a low dose of sorafenib. A CR was obtained following eight months of oral administration and there was no recurrence for a further eight months following discontinuation. In the present case, a CR was achieved after two years of oral administration and no recurrence has been detected for one year since discontinuation.

Several hypotheses concerning the maintenance of a CR following the discontinuation of sorafenib have been discussed. Wang *et al*(10) considered it most likely due to the uniqueness of the tumor biopsy, i.e., activated by a single or few pathway(s) that was/were completely blocked by sorafenib.

Alternatively, So *et al*(9) suggested that the tumor was highly dependent for survival on one or more of the receptor tyrosine kinases that are inhibited by sorafenib. The mechanism is unclear, but there may be specific molecular level features of HCC cases in which CR is maintained following the discontinuation of sorafenib that differ from those of other cases.

In the present patient, sorafenib was discontinued four months after the judgment of a CR, whereas the drug was withdrawn at almost the same time as the diagnosis of a CR in two of the previous cases (4,10) and after one month in one case (9). In patients with renal cell carcinoma (RCC) treated with sorafenib, Johannsen *et al*(14) observed that recurrent or new metastatic lesions developed following discontinuation of the drug in five out of 12 patients who achieved a CR. A further accumulation of cases is required to understand the appropriate timing of the discontinuation of sorafenib after a CR is achieved.

In conclusion, the present study described a case of advanced HCC with PVTT that showed a CR following treatment with low-dose sorafenib (400 mg once daily) and in which this CR was maintained for approximately one year after treatment was discontinued. Tumors may recur due to the discontinuation of treatment, and the appropriate timing of sorafenib discontinuation requires further investigation.

## References

[1] Parkin DM, Bray F, Ferlay J, Pisani P (2005). Global cancer statistics, 2002. CA Cancer J Clin.

[2] Llovet JM, Ricci S, Mazzaferro V (2008). Sorafenib in advanced hepatocellular carcinoma. N Engl J Med.

[3] Cheng AL, Kang YK, Chen Z (2009). Efficacy and safety of sorafenib in patients in the Asia-Pacific region with advanced hepatocellular carcinoma: a phase III randomized, double-blind, placebo-controlled trial. Lancet Oncol.

[4] Inuzuka T, Nishikawa H, Sekikawa A (2011). Complete response of advanced hepatocellular carcinoma with multiple lung metastases treated with sorafenib: a case report. Oncology.

[5] Chan A, Tan EH (2011). How well does the MESTT correlate with CTCAE scale for the grading of dermatological toxicities associated with oral tyrosine kinase inhibitors?. Support Care Cancer.

[6] Edeline J, Boucher E, Rolland Y (2012). Comparison of tumor response by Response Evaluation Criteria in Solid Tumors (RECIST) and modified RECIST in patients treated with sorafenib for hepatocellular carcinoma. Cancer.

[7] Wilhelms S, Carter C, Lynch M (2006). Discovery and development of sorafenib: a multikinase inhibitor for treating cancer. Nat Rev Drug Discov.

[8] Shiozawa K, Watanabe M, Kikuchi Y, Kudo T, Maruyama K, Sumino Y (2012). Evaluation of sorafenib for hepatocellular carcinoma by contrast-enhanced ultrasonography: a pilot study. World J Gastroenterol.

[9] So BJ, Bekaii-Saab T, Bloomston MA, Patel T (2008). Complete clinical response of metastatic hepatocellular carcinoma to sorafenib in a patient with hemochromatosis: a case report. J Hematol Oncol.

[10] Wang SX, Byrnes A, Verma S, Pancoast JR, Rixe O (2010). Complete remission of unresectable hepatocellular carcinoma treated with reduced dose of sorafenib: a case report. Target Oncol.

[11] Kudo M, Ueshima K (2010). Positioning of a molecular-targeted agent, sorafenib, in the treatment algorithm for hepatocellular carcinoma and implication of many complete remission cases in Japan. Oncology.

[12] Sacco R, Bargellini I, Gianluigi G (2011). Complete response for advanced liver cancer during sorafenib therapy: case report. BMC Gastroenterol.

[13] Li Q, Xu B, Fu L, Hao XS (2006). Correlation of four vascular specific growth factors with carcinogenesis and portal vein tumor thrombus formation in human hepatocellular carcinoma. J Exp Clin Cancer Res.

[14] Johannsen M, Flörcken A, Bex A (2009). Can tyrosine kinase inhibitors be discontinued in patients with metastatic renal cell carcinoma and a complete response to treatment? A multicenter, retrospective analysis. Eur Urol.

